# A One Health approach revealed the long-term role of *Mycobacterium caprae* as the hidden cause of human tuberculosis in a region of Spain, 2003 to 2022

**DOI:** 10.2807/1560-7917.ES.2023.28.12.2200852

**Published:** 2023-03-23

**Authors:** Miguel Martínez-Lirola, Marta Herranz, Sergio Buenestado Serrano, Cristina Rodríguez-Grande, Eva Dominguez Inarra, Jose Antonio Garrido-Cárdenas, Ana María Correa Ruiz, María Pilar Bermúdez, Manuel Causse del Río, Verónica González Galán, Julia Liró Armenteros, Jose María Viudez Martínez, Silvia Vallejo-Godoy, Ana Belén Esteban García, María Teresa Cabezas Fernández, Patricia Muñoz, Laura Pérez Lago, Darío García de Viedma

**Affiliations:** 1 Unidad de Gestión de Laboratorios, UGMI, Complejo Hospitalario Torrecárdenas, Almería, Spain; 2 Servicio de Microbiología Clínica y Enfermedades Infecciosas, Hospital General Universitario Gregorio Marañón, Madrid, Spain; 3 Instituto de Investigación Sanitaria Gregorio Marañón (IiSGM), Madrid, Spain; 4 CIBER Enfermedades Respiratorias (CIBERES), Madrid, Spain; 5 Laboratorio de Producción y Sanidad Animal de Córdoba, Consejería de Agricultura, Ganadería, Pesca y Desarrollo Sostenible, Córdoba, Spain; 6 Department of Biology and Geology, Universidad de Almería, Almería, Spain; 7 Microbiology Unit, Costa del Sol Hospital, Marbella, Málaga, Spain; 8 Servicio de Microbiología, Hospital Regional de Málaga, Málaga, Spain; 9 Servicio de Microbiología, Hospital Universitario Reina Sofía, Córdoba, Spain; 10 Servicio de Microbiología, Hospital Virgen del Rocío, Sevilla, Spain; 11 Servicio de Enfermedades Infecciosas y Microbiología, Hospital Universitario Virgen Macarena, Sevilla, Spain; 12 Oficina Comarcal Agraria de Huércal-Overa, Almería, Spain; 13 Servicio de Medicina Preventiva, Hospital Universitario Poniente de Almería, Almería, Spain; 14 Servicio de Análisis de Ácidos Nucleicos, Servicios Centrales de Investigación de la Universidad de Almería, Almería, Spain; 15 Departamento de Medicina, Universidad Complutense, Madrid, Spain

**Keywords:** Tuberculosis, *M. caprae*, WGS, One Health

## Abstract

**Introduction:**

*Mycobacterium caprae* is a member of the *Mycobacterium tuberculosis* complex (MTBC) not routinely identified to species level. It lacks specific clinical features of presentation and may therefore not be identified as the causative agent of tuberculosis. Use of whole genome sequencing (WGS) in the investigation of a family microepidemic of tuberculosis in Almería, Spain, unexpectedly identified the involvement of *M. caprae*.

**Aim:**

We aimed to evaluate the presence of additional unidentified *M. caprae* cases and to determine the magnitude of this occurrence.

**Methods:**

First-line characterisation of the MTBC isolates was done by MIRU-VNTR, followed by WGS. Human and animal *M. caprae* isolates were integrated in the analysis.

**Results:**

A comprehensive One Health strategy allowed us to (i) detect other 11 *M. caprae* infections in humans in a period of 18 years, (ii) systematically analyse *M. caprae* infections on an epidemiologically related goat farm and (iii) geographically expand the study by including 16 *M. caprae* isolates from other provinces. Integrative genomic analysis of 41 human and animal *M. caprae* isolates showed a high diversity of strains. The animal isolates’ diversity was compatible with long-term infection, and close genomic relationships existed between isolates from goats on the farm and recent cases of *M. caprae* infection in humans.

**Discussion:**

Zoonotic circulation of *M. caprae* strains had gone unnoticed for 18 years. Systematic characterisation of MTBC at species level and/or extended investigation of the possible sources of exposure in all tuberculosis cases would minimise the risk of overlooking similar zoonotic events.

**Key public health message**

**What did you want to address in this study?**

*Mycobacterium caprae* is a pathogen infecting wild and domestic animals. It belongs to the same bacterial group as *M. tuberculosis* that causes tuberculosis in humans. Human cases of tuberculosis are not usually investigated to species level. After detecting a family cluster of tuberculosis caused by *M. caprae*, we wanted to know how frequently undetected infections with this bacterium occur in humans.

**What have we learnt from this study?**

We did a systematic genomic analysis of other human tuberculosis cases in a wider geographical area and compared this with bacterial isolates obtained from epidemiologically related animals. This allowed us to discover and characterise in depth an extensive endemic zoonosis involving *M. caprae* that had remained unnoticed for 18 years.

**What are the implications of your findings for public health?**

A proportion of tuberculosis cases in our population were caused by a bacterium that can infect a range of animals, *M. caprae*. Identification and characterisation of *M. caprae* must be mandatory when a person is diagnosed with tuberculosis, and veterinarian regulations for infection control in goats must be re-evaluated.

## Introduction

*Mycobacterium caprae* is a member of the *Mycobacterium tuberculosis* complex (MTBC), which was not recognised as a separate species until 2003 [1]. It is the main causative agent of tuberculosis (TB) in goats, but also infects other domestic and wild animals [2,3]. *Mycobacterium caprae* does not present any unique features that enable it to be identified in routine microbiological diagnosis, as is the case with *M. bovis*, which is intrinsically resistant to pyrazinamide. This means that *M. caprae* may remain unidentified at species level, since most diagnostic laboratories only test as far as MTBC. At the clinical level, most human cases of *M. caprae* TB present with pulmonary involvement [4] and without special features, which also explains why we may fail to identify *M. caprae* as the causative agent. When specific approaches are applied to identify this species with certainty, its representation has been greater than expected; this was the case in Germany, where *M. caprae* was identified in a 1:3 ratio with respect to *M. bovis* [5], and in Spain, where among 110 zoonotic TB cases in the period 2004 to 2007, 89 involved *M. bovis* and 21 *M. caprae* [6].

Our study was centred on the province of Almería, south-eastern Spain, where, for the past 18 years, we have run a population-based, systematic molecular epidemiology analysis based on mycobacterial interspersed repetitive unit-variable number tandem repeat (MIRU-VNTR) typing to guide the TB control programme in its interventions. Genomic analysis is also applied in this setting when VNTR resolution is insufficient to clarify TB transmission dynamics. Here we present a comprehensive One Health study that arose from the analysis of one such cluster that required genomic resolution.

### Methods

### MIRU-VNTR

We performed MIRU-VNTR typing as described elsewhere [7]. Briefly, PCR was performed to amplify 24 loci in DNA samples using eight triplex PCR reactions. The standard protocol was followed for six triplex reactions, using the multiplex PCR kit (Qiagen, Hilden, Germany). For the remaining two, PuReTaq Ready-To-Go PCR beads (GE Healthcare, Chicago, United States (US)) were used, adding 0.5 µM of each primer and 3% dimethyl sulfoxide (DMSO). Sizing of PCR fragment was done by capillary electrophoresis (3130 Genetic Analyzer, Applied Biosystems, Waltham, US). Alleles were assigned with GeneMapper 4.0 (Thermo Scientific, Waltham, US).

### Retrospective compilation of human *Mycobacterium caprae* isolates from other provinces in Andalusia

We compiled the *M. caprae* isolates identified by performing the DNA-strip assay, GenoType MTBC (Bruker, Billerica, US) from one hospital in Málaga (2005 to 2022), one hospital in Marbella (2010 to 2022), two hospitals in Sevilla (2005 to 2022, and 2011 to 2022) and one from Córdoba (2017 to 2022). Fresh passages of the corresponding frozen strains were sent to the Complejo Hospitalario Torrecárdenas (Almería) and the Hospital Gregorio Marañón laboratories (Madrid) to perform 24-locus MIRU-VNTR and WGS, respectively.

### Identification of infected animals

The goats on the farm owned by the couple under study were subjected to the tuberculin skin test. The skin test was carried out according to Royal Decree 2611/1996, of 20 December 1996, which regulates the national programmes for the eradication of animal diseases, and the Order of 22 June 2018, which develops the qualification standards against TB for goat farms in Andalusia and develops the rules for the implementation of the programme for surveillance, prevention, control and eradication of animal diseases in Andalusia. A simple tuberculin test was performed. Briefly, the injection site was shaved and cleaned, the thickness of the skin was measured with a cutimeter and recorded, the corresponding dose of tuberculin was injected. At 72 h (± 4 h) after the injection, the thickness of the skin was measured again, and the result was recorded for later comparison with the first measurement and interpretation. In addition, at the time of the second reading, a clinical assessment of the animal was made. The skin test was performed by veterinarians authorised by the “Rumial” Livestock Health Defence Group to which the farm belongs, who are in charge of carrying out the health programmes of their associates. These professionals had previously received a training course on the diagnosis of TB.

Animals positive in the tuberculin skin test were slaughtered. A selection of them were randomly chosen for necropsy. At necropsy, lung nodules and lymph nodes (mediastinal and tracheobronchial), as well as other tissues with macroscopic TB-like lesions, were pooled, decontaminated (N-acetyl-l-cysteine–sodium hydroxide (NALC-NaOH)), inoculated into mycobacteria growth indicator tube (MGIT) medium and incubated in the MGIT 960 instrument until flagged positive, or for a maximum of 6 weeks. All positive MGIT tubes were tested for the presence of MTBC using the VetMAX *M. tuberculosis* Complex kit (Applied Biosystems) based on probe hybridisation of the IS*6110* insertion sequence.

### Whole genome sequencing

Whole genome sequencing was performed on purified genomic DNA from culture isolates as previously described [8]. The libraries were prepared using the Nextera XT kit (Illumina, San Diego, US) and libraries were run in a MiSeq device (2 × 151 bp) which generated an average per base coverage of 151.37×. Fastq files with the raw data were deposited under accession number PRJEB56608 (ENA) (http://www.ebi.ac.uk).

Sequence analyses were done using a homemade pipeline deposited in Git-Hub (https://github.com/MG-IiSGM/autosnippy). Briefly, the pipeline went through the following steps: (i) species identification with Kraken2 v2.1.2 and Mash v2.3; (ii) mapping and variant calling performed with snippy v4.6.0, which maps using Burrows–Wheeler alignment (BWA-MEM v0.7.17) and variant calling with freebayes v1.3.2, using the *M. caprae* strain Allgaeu (NZ_CP016401.1) as reference; (iii) variant annotation with SnpEff v5.1; and (iv) occasional recalibration of low-coverage positions using joint variant calling. Highly polymorphic and repetitive regions, phages and PE/PPE regions were removed from the final single nucleotide polymorphism (SNP) distance calculation and annotation. We also excluded SNPs located close to indels or in areas with a higher-than-expected number of calls (≥ 3 SNPs within 10 bp of each other).

Alignments and SNP variants were visualised and checked with the integrative genomics viewer (IGV) programme [9]. Median-joining networks of genomic relationships were constructed from the SNP matrix generated with NETWORK 5.0. Median vectors were defined when the distribution of SNPs indicated the existence of a non-sequenced node corresponding to a genotype that had not been sampled in the cluster.

The in silico spoligotyping and the extraction of the standard international type (SIT) number of the WGS data was performed with the open-acess SpoTyping tool (https://github.com/xiaeryu/SpoTyping-v2.0).

## Results

### Family cluster of tuberculosis

In 2020, a cluster of two TB cases sharing identical MIRU-VNTR patterns was detected in a universal molecular epidemiology programme that had been running in Almería since 2003. The cluster involved a married couple of goat farmers diagnosed with TB in January and February 2020, respectively (Case 1478 (Farmer A), and their spouse, Case 2672 (Farmer B)) (Table). An interpretative dilemma arose when trying to obtain the most likely epidemiological explanation for this cluster. 

**Table:** Tuberculosis cases involving *Mycobacterium caprae* **,** Andalusia, 2003–2022 (n = 30)

ID patient	Year	Origin	Risk factor for *M. caprae* infection^a^	Mirutyping loci																							
				M02	M20	M23	M24	M27	M39	M4	M26	M40	M10	M16	M31	M42	M43	ETRA	47	52	53	Qub 11b	1995	Qub 26	M46	M48	M49
118	2003	Spain (Almería)	Unknown	2	2	4	2	3	2	4	5	2	3	4	**5**	3	5	4	4	1	2	2	1	2	3	3	2
1401	2011	Spain (Almería)	Unknown	2	2	4	2	3	2	4	5	2	3	4	**5**	3	5	4	4	1	2	2	1	2	3	3	2
1478.1 (Farmer A, first episode)	2011	Spain (Almería)	Professional exposure	2	2	4	2	3	2	4	5	2	3	4	**5**	3	5	4	4	1	2	2	1	2	3	3	2
1478.2 (Farmer A, second episode)	2020	Spain (Almería)	Professional exposure	2	2	4	2	3	2	4	5	2	3	4	**2**	3	5	4	4	1	2	2	1	2	3	3	2
2672 (Farmer B)	2020	Spain (Almería)	Professional exposure	2	2	4	2	3	2	4	5	2	3	4	**2**	3	5	4	4	1	2	2	1	2	3	3	2
2142 (sibling A)	2016	Spain (Almería)	Consumer of unpasteurised milk	2	2	4	2	3	2	4	5	2	3	4	**2**	3	5	4	4	1	2	2	1	2	3	3	2
2319 (sibling B)	2017	Spain (Almería)	Consumer of unpasteurised milk	2	2	4	2	3	2	4	5	2	3	4	**2**	3	5	4	4	1	2	2	1	2	3	3	2
1300	2010	Spain (Almería)	Unknown	2	2	4	2	3	2	4	5	**1**	3	4	**5**	3	5	4	4	1	2	2	1	2	3	3	2
1485	2011	Mauritania	Exposure to goats	2	2	4	2	3	2	4	5	2	3	4	**5**	3	5	**5**	4	1	2	**4**	1	2	3	3	2
1986	2015	Spain (Almería)	Professional exposure	2	2	4	2	3	2	4	5	2	3	4	**2**	**1**	5	4	4	1	2	**8**	1	2	3	3	2
2127	2016	Mali	Exposure to cattle	2	2	4	2	3	2	4	5	2	3	4	**5**	3	5	**5**	4	1	2	**4**	1	2	3	3	2
2309	2017	Spain (Almería)	Professional exposure	2	2	4	2	3	2	4	5	2	3	4	**2**	3	5	4	4	1	2	2	1	2	3	**2**	2
2486	2019	Spain (Almería)	Unknown	2	2	4	2	3	2	4	5	2	3	4	**5**	3	5	**5**	4	1	2	**4**	1	2	3	3	2
2929	2021	Spain (Almería)	Professional exposure	2	2	4	2	3	2	4	5	2	3	4	**2**	3	5	4	4	1	2	2	1	2	3	**2**	2
3006	2017	Spain (Malaga)	Professional exposure	2	2	4	2	3	2	4	5	2	3	4	**5**	3	5	4	4	1	2	2	1	2	3	3	2
3007	2018	Spain (Malaga)	Professional exposure	2	2	4	2	3	2	4	5	2	3	4	**2**	3	5	4	4	1	2	2	1	2	3	3	2
3008	2018	Spain (Malaga)	Professional exposure	2	2	4	2	3	2	4	5	2	3	4	**5**	3	**4**	4	4	1	2	2	1	2	3	**2**	2
3010	2019	Western Sahara	Unknown	2	2	4	2	3	2	4	5	2	3	4	**5**	3	**4**	4	4	1	2	2	1	2	3	**2**	2
3011	2021	Spain (Sevilla)	Professional exposure	2	2	**3**	2	3	2	4	5	2	3	4	**5**	3	5	4	4	1	2	2	1	2	3	3	2
3012	2021	Spain (Malaga)	Professional exposure	2	2	4	2	3	2	4	5	2	3	4	**5**	3	5	4	4	1	2	2	1	2	3	3	2
3013	2021	Spain (Malaga)	Professional exposure	2	2	4	2	3	2	4	5	2	3	4	**5**	3	5	**5**	4	1	2	4	1	2	3	3	2
3015	2015	Spain (Sevilla)	Unknown	2	2	4	2	3	2	4	5	2	3	4	**2**	3	5	4	4	1	2	2	1	2	3	3	2
3016	2017	Spain (Sevilla)	Unknown	2	2	4	2	3	2	4	5	2	3	4	**2**	3	5	4	4	1	2	2	1	2	3	3	2
3040	2022	Spain (Córdoba)	Professional exposure	2	2	4	2	3	2	4	5	2	3	4	**2**	3	5	4	4	1	2	2	1	2	3	3	2
3041	2019	Spain (Córdoba)	Exposure to goats	2	2	4	2	3	2	4	5	2	3	4	**2**	3	5	4	4	1	2	2	1	2	3	3	2
3045	2011	Lithuania	Unknown	2	2	4	2	3	2	4	5	2	3	4	**5**	3	5	4	4	1	2	2	1	2	3	3	2
3046	2013	Morocco	Unknown	2	2	4	2	3	2	4	5	2	**6**	4	**5**	2	5	4	4	1	2	**4**	1	2	3	**2**	2
3048	2018	Spain (Sevilla)	Professional exposure	2	2	4	2	3	2	4	5	2	3	4	**2**	3	5	4	4	1	2	2	1	2	3	3	2
3049	2020	Spain (Sevilla)	Unknown	2	2	4	2	3	2	4	5	2	3	4	**2**	3	5	4	4	1	2	2	1	2	3	3	2
3050	2018	Spain (Sevilla)	Professional exposure	2	2	NA	2	3	2	4	5	2	3	4	**2**	3	5	4	4	1	2	2	1	2	3	3	2

NA: not available.

^a^ We differentiate between professional exposure and non-systematic exposure (e.g. farm visitors).

Bold numbers represent the allele diversity identified in certain MIRU-VNTR loci with respect to the majority allele among the isolates in the study.

On the one hand, their MIRU-VNTR pattern had also been identified in another two cases, diagnosed in 2016 and 2017 (Cases 2142 and 2319, Table). On the other hand, Case 1478 had had a previous TB episode 9 years earlier, where the VNTR pattern differed by only one single locus variant (SLV) from the current VNTR pattern and was shared by another two cases diagnosed in 2003 and 2011 (Cases 118 and 1401). Considering these data as a whole, we studied two possible interpretations for the infection in the farm couple in 2020: either they had recently been infected with a circulating strain (the one which had infected Cases 2142 and 2319 in the past), or Case 1478 could have experienced a recent reactivation of their previous infection, generating an SLV by microevolution and subsequently infecting their spouse.

**Figure 1:**
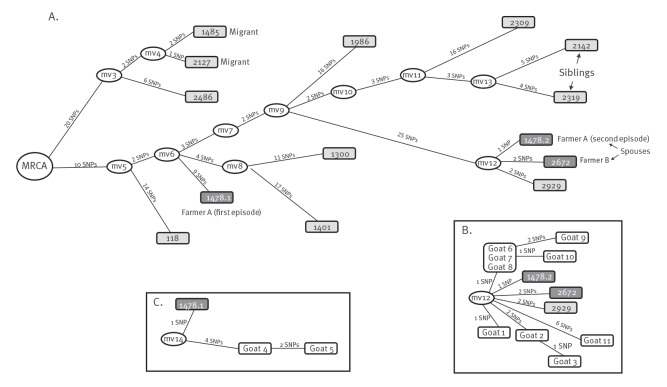
Network of relationships between the *Mycobacterium caprae* isolates from human cases, based on whole genome sequence, Almería, 2003–2021 (n = 14)

MRCA: most recent common ancestor; mv: median vector; SNP: single nucleotide polymorphism.

Seeking additional support to clarify which of the two interpretations was correct, we performed WGS on the two isolates taken from the two farmers in 2020 and the one from Farmer A in 2011. The WGS data revealed that all three isolates corresponded to *M. caprae*, not to *M. tuberculosis*. Furthermore, the isolates from 2020 differed from each other by 3 SNPs. Finally, the two isolates from Farmer A in years 2011 and 2020 differed from each other by 40 SNPs (Figure 1A).

Panel A shows human isolates from Almería; panels B and C show isolates from goats together with those human isolates which were closely related to them.

Numbers in boxes correspond to the isolates’ reference numbers. Dark boxes: farmers; siblings and migrants are specified with labels close to the corresponding box). Ellipses: nodes in the network corresponding to theoretical isolates which were not sampled. Genomic distances (number of SNPs) between the isolates are indicated on the linking lines. Length of the linking lines length are not to scale.

### Retrospective tracking for *Mycobacterium caprae*

In view of these unexpected findings, we undertook a search for all isolates in the Almería population sharing identical or similar MIRU-VNTR types (allowing single/double loci variations) with the two farm cases under study and identified another seven cases from the period 2010 to 2021), in addition to the four cases previously identified (Table). The WGS analysis indicated that all 11 isolates were *M. caprae*, demonstrating the unsuspected presence of this species in human infections over 18 years. Sociodemographic and clinical characteristics of these cases are appended in the Supplement.

Genomic analyses showed a great diversity of strains (pairwise SNP distances between each of two isolates was 3–70 SNPs, Figure 1A); most of them unrelated and positioned on a separate branch in the network. Among the few exceptions sharing a branch and closely related, we found (i) the 2020 isolates from the two farmers and Case 2929 (year 2021) and (ii) isolates from two migrants (Cases 1485 and 2127 in 2011 and 2016; Figure 1A).

We attempted to interview all 13 cases identified to obtain information on contact with livestock. Unfortunately, five were deceased and of the remainder, only six agreed to be interviewed. Consistent with our findings, the couple owned a goat farm, which meant constant exposure to animals. Case 2929 also owned a goat farm, close to the couple’s farm (6.6 km apart). Case 2309 had worked as a slaughterer, and Cases 2142 and 2319 were siblings (diagnosed when they were in their mid-30s and late 20s) who reported that they had consumed unpasteurised milk in their childhood. The location in the genomic network of the two sibling cases was compatible with common exposure in the past (median vector mv13). The two isolates obtained from the two migrants diagnosed in 2011 and 2016 were also closely related (Cases 1485 and 2127; 3 SNPs), although these individuals had arrived from two different countries in the Sahel region. 

### Integrative One Health study

A more complete understanding of the health problem posed by the unexpected identification of human TB cases involving *M. caprae* required shifting to a One Health strategy, which meant testing the 363 goats (16 goats > 9 years-old) on the farm owned by the couple. We tested these goats by tuberculin skin test in June 2021 to evaluate infection, and 121 animals (33.3%) were tuberculin-positive.

We identified macroscopic TB-like lesions in 13 (11%) of the positive slaughtered animals. Among the positive animals, we randomly selected 24 for necropsy and could isolate *M. caprae* from 11 of them. The 11 *M. caprae* isolates obtained from the goats were also analysed by WGS and the data were integrated with those from the human isolates in the same genomic network. Nine of the animal isolates were located on the same branch as the 2020 isolates from the farm owners and all were distributed on branches sharing a common node (Figure 1B). The two remaining animal isolates were found sharing a node with the 2011 isolate collected from Farmer A (Figure 1C).

### Geographically expanded search for *Mycobacterium caprae*

Finally, in order to expand our geographical analysis beyond Almería by adding data from other Andalusian provinces, we included a further 24 *M. caprae* isolates identified in humans (years 2011–2022) at the hospitals in Andalusia where *M. caprae* was identified to species level. Sociodemographic and clinical characteristics of these cases are appended in the Supplement. In 27 cases (23 men, 4 women, age range: 24–65 years), we were able to find potential risk factors for *M. caprae* infection: 13 occupational exposures (seven workers on goat farms, three on a cattle farm, two slaughterers and one person who handled animal remains to produce fodder), two family relationships with cattle owners and five migrants from countries where consumption of non-pasteurised milk is likely (four from the Sahel region and one from Sub-Saharan Africa; one reported that they had consumed unpasteurised milk).

**Figure 2:**
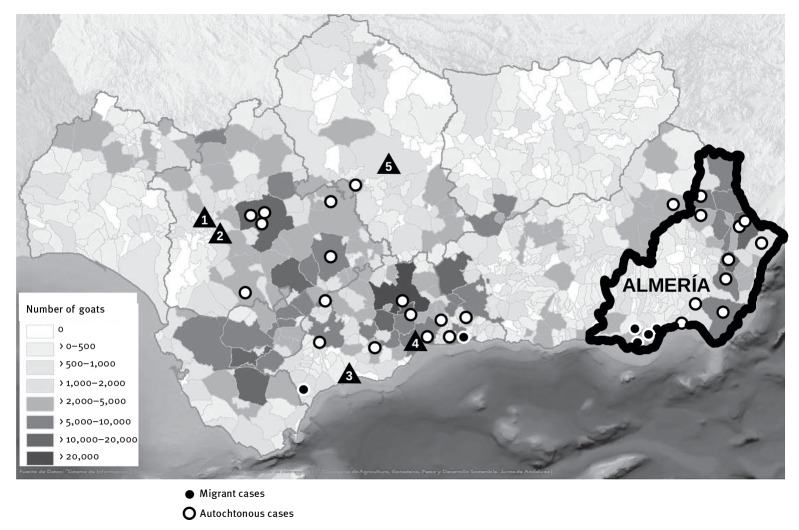
*Mycobacterium caprae* human cases, by place of residence, and density of goats (year 2020) on the farms located in the region, Andalusia, 2003–2022 (n = 33)

We positioned the *M. caprae* cases (by place of residence) in the map of the Andalusia region, together with the distribution of goat farms and the number of animals on the farms. The distribution of human cases coincided mostly with the areas in Andalusia with the highest density of goats on farms (Figure 2).

Almería is highlighted with bolder province limits. All diagnosed cases are included in the map, regardless of whether their isolates were available for analysis. Circles: human cases; white dots: authochtonous cases; black dots: migrant cases. Triangles: the cities with hospitals performing intra-MTBC species identification: 1 = Hospital Universitario Virgen del Rocío (Sevilla), 2 = Hospital Universitario Virgen Macarena (Sevilla), 3 = Hospital Costa del Sol (Malaga), 4 = Hospital Universitario Carlos Haya (Malaga) and 5 = Hospital Universitario Reina Sofía (Córdoba).

Source for the data: SIGEAM, Consejería de Agricultura, Ganadería, Pesca y Desarrollo sostenible, Junta de Andalucía.

**Figure 3:**
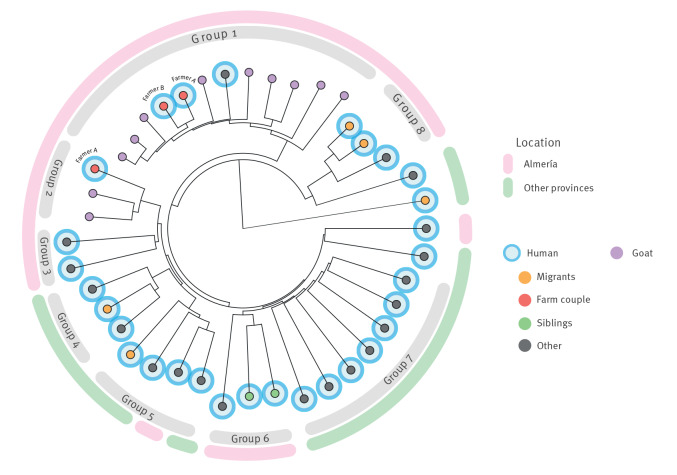
Phylogenetic tree of the *Mycobacterium caprae* sequences from Almería (human cases and goats) and other provinces (only human cases), Andalusia, 2003–2022 (n = 41)

**Figure 4:**
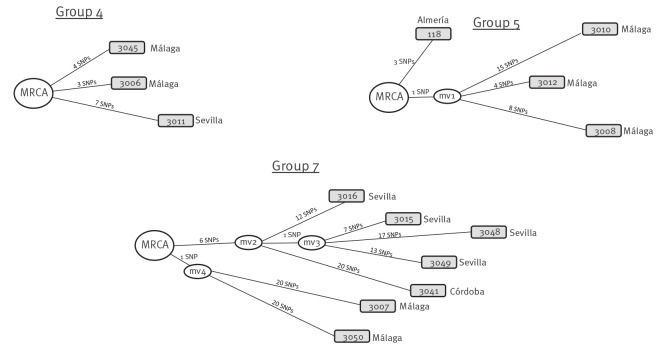
Network of relationships between *Mycobacterium caprae* sequences from humans in phylogenetic groups 4, 5 and 7, Andalusia, 2003–2022 (n = 14)

MRCA: most recent common ancestor; mv: median vector; SNP: single nucleotide polymorphism.

When we included all the new isolates from Andalusia in which genomic analysis was possible with those previously obtained from Almería (from humans and goats), we obtained a phylogenetic distribution in eight groups (Figure 3). Groups 1, 2, 3, 6 and 8 only included isolates from Almería, groups 4 and 7 included isolates from other provinces but not from Almería, and the only group with both isolates from Almería and other provinces was Group 5. The genomic pairwise distances measured within the groups 4, 5 and 7, which included the isolates from other provinces, were 7–11, 8–23 and 20–47 (Figure 4), indicating a wide diversity between the strains included.

Phylogenetic tree done with Microreact (https://microreact.org), using the genomic pairwise SNP distances between the isolates. Each dot corresponds to an isolate. Dots surrounded by a ring correspond to human isolates; the colours for the dot indicates which isolates correspond to the farmers, siblings or migrants. Outer rings indicate the isolates from Almería (pink) or from other provinces (green). Isolates are included in groups (grey, groups 1–8), supported on their inclusion in the same branch of the tree.

Numbers in boxes correspond to the patient reference number. Ellipses: nodes in the network corresponding to hypothetical isolates which were not sampled. Genomic distances (number of SNPs) between the isolates are indicated on the linking lines. Length of the linking lines length are not to scale.

## Discussion

A small family cluster of TB cases was the starting point for the discovery of the hidden presence of *M. caprae* causing human infections in Andalusia in the south of Spain. It was only the need to perform WGS to increase the discriminatory power of VNTR analysis to determine the SNP distance between the isolates in question and to shed light on the cluster that revealed that the isolates in fact were *M. caprae*. This discovery led us to identify, among the TB cases diagnosed in the same hospital in Almería, 11 other cases infected with *M. caprae* (suspected by their MIRU-VNTR patterns compatible with *M. caprae* and finally confirmed by WGS) that had initially been attributed to *M. tuberculosis*. These findings account for the undetected presence of *M. caprae* infections in the population of Almería over the past 18 years.

Our findings should not be regarded as an event restricted to a local area, as it may well be occurring in other settings. Only five of the 17 mycobacterial laboratories in hospitals belonging to the Andalusian Public Network routinely differentiate MTBC species (another two have performed it only since 2020 and 2022): It has been suggested that *M. caprae* plays an emerging role as the pathogen responsible for animal TB, which has already overtaken *M. bovis* in certain countries [10]. One study in Switzerland reported that *M. caprae* had been present on a farm and gone undetected for 15 years, even though the country was officially bovine TB (bTB)-free during that period [11]. In another officially bTB-free region in Italy [12], supported by negative results of tuberculin skin testing of animals every 3 years, bTB due to *M. caprae* was detected when the diagnostic approach was extended to interferon γ-based testing or direct post-mortem analysis. A recently described outbreak on a farm for rabbits [2], a species not previously included among those potentially infected by *M. caprae*, suggests that the risks of exposure may be wider than previously considered.

Like *M. bovis*, the main routes for acquiring *M. caprae* infection are consumption of unpasteurised dairy products and/or close contact with infected animals. The epidemiological investigation triggered by our findings identified that the couple who initiated the study owned a goat farm. The discovery of the farmers’ occupational exposure to goats in or study led us to adopt a One Health approach and to integrate data from both animal and human *M. caprae* infections in order to obtain a better understanding of this event. Spain, like other countries in the European Union, has a bovine TB eradication programme in place, but does not include systematic testing of goats, except in certain autonomous regions that have a high density of goat flocks in close contact with cattle herds. Therefore, *M. caprae* infections in animals are probably underdiagnosed. Data from Spain indicate that 7.4% of bTB is linked to *M. caprae* and that 0.3% of human TB cases between 2004 and 2007 were due to *M. caprae* [13]. These figures probably underestimate the true frequency of infections with this pathogen. To obtain information on the infection status of the studied farm, we reported our results to the veterinary authorities, who intervened and detected a high rate of tuberculin reactivity among the animals. The *M. caprae* isolates obtained from a selection of the slaughtered positive animals proved invaluable for completing the integrative analysis of animal and human infections.

Having complemented the collection of *M. caprae* isolates in Almería with the animal isolates from the farm, we extended our study sample to *M. caprae* isolates detected in humans in the five Andalusian hospitals that routinely identified *M. caprae* to species level. Most of these cases were related to exposure to animals or people born in countries where consumption of unpasteurised milk is common (we must acknowledge as a limitation that consumption of hunted venison was not included among the questions included in the epidemiological investigations). In most patients, TB was respiratory, suggesting that air-mediated exposure to infected animals was more frequent than consumption of contaminated products.

All human and animal isolates were analysed by WGS and data were integrated in a single analysis to detect potential relationships between them. Firstly, we observed wide SNP-based diversity among the human isolates, not only among those from different provinces, but also those from the same province, suggesting that many different strains were responsible for human infections.

Closer genomic relationships were found only between two migrants and two siblings in Almería. The migrants (from two different countries in the Sahel region) had lived in the same village in Spain for several years before their diagnosis. The standard interview with one of them did not reveal any exposure to animals. It was necessary to perform extensive interviews trying to find some non-obvious exposure to goats, and it finally emerged that before diagnosis, when the person was severely immunocompromised, they had passed daily next to a flock of goats and sheep when going to their workplace in a greenhouse. Small flocks in that area are frequently fed with vegetal remnants from greenhouses, in the narrow corridors between and even inside them, and the case frequently consumed meat from these local flocks (mostly not controlled by the veterinarian authorities). The other case was in charge of purchasing the livestock from those local flocks to prepare and cook them for a public celebration (in which livestock are slaughtered and distributed to be cooked).

Interestingly, the position in the genomic network of the siblings who had consumed unpasteurised milk in childhood was compatible with a common past exposure and a subsequent independent within-patient evolution of the strain. The number of differential SNPs identified in each sibling (4 and 5 SNPs) was consistent with the time elapsed between exposure in their childhood and diagnosis (years 2016 and 2017) and the generally assumed acquisition rate for MTBC (0.3–0.5 SNPs/year).

The isolates from the farm couple were exceptionally closely related both to each other and with respect to the animal isolates obtained from their farm. These findings proved the involvement of this farm in the zoonotic event under study. Another human isolate from an individual who owned a goat farm in the vicinity was also genomically closely related. Wild boars have recently been pointed out as the infectious link between goat herds in Catalonia [14]; however, this link is unlikely in our case, because the couple applied intensive farming practices and the animals did not leave the farm.

Interestingly, we observed some diversity among the animal isolates. Only three goats were infected with identical strains, while the rest showed a limited number of SNPs, despite the fact that most of them shared the same branch in the network. Limited diversity was also observed between the two isolates from the couple. The isolate from Farmer A (i) had one unique SNP that was not shared by their spouse’s isolate, and (ii) did not harbour two SNPs which were unique to their spouse’s isolate. These observations suggest that this was a long-term infected farm in which diversity had been acquired by microevolution, and that different clones were responsible for each of the two human infections. Microevolution within infected herds has been demonstrated both for *M. bovis* [15] and *M. caprae* [11,16]. The suspected long-term infection status of the farm was reinforced by another unexpected finding. Two isolates from the goats slaughtered in 2021 were positioned in the network at a considerable distance from most other goat isolates and were instead positioned closer to the isolate obtained from Farmer A in 2011. This suggests the probable co-existence on the farm of microevolved variants of the strain that had caused the first exposure of Farmer A at least 10 years previously. This observation is supported by the presence in 2021 of > 9-year-old goats; therefore, animals potentially infected in 2011 could still have been present in this herd in 2020.

We should remember that an investigation into exposure to infected animal was not performed for Farmer A’s episode in 2011, as *M. tuberculosis* was assumed to be the causative agent. From our findings, it seems essential to include professional risk to trace the origin of any MTBC infection whenever TB is confirmed in a patient, even when the identification to species level is not done.

## Conclusion

Whole genome sequencing analysis was instrumental in revealing the hidden role of *M. caprae*, first in one particular cluster and then in an otherwise unsuspected long-term zoonotic event that had gone unnoticed for 18 years, involving a high diversity of *M. caprae* strains infecting humans and undetected long-term infection of epidemiologically related goats. In the authors’ opinion, identification and characterisation of *M. caprae* should always be undertaken when a person is diagnosed with TB, exposure to cattle should be included in the systematic epidemiological investigation of any new TB case and veterinarian regulations for infection control in goats should be re-evaluated.

## Supplementary Data

25000 Supplement
